# IL-23 Contributes to *Campylobacter jejuni*-Induced Intestinal Pathology *via* Promoting IL-17 and IFNγ Responses by Innate Lymphoid Cells

**DOI:** 10.3389/fimmu.2020.579615

**Published:** 2021-01-06

**Authors:** Xi Jing, Anna A. Korchagina, Sergey A. Shein, Wayne T. Muraoka, Ekaterina Koroleva, Alexei V. Tumanov

**Affiliations:** ^1^ Department of Microbiology, Immunology and Molecular Genetics, University of Texas Health Science Center at San Antonio, San Antonio, TX, United States; ^2^ Department of Gastroenterology, Third Xiangya Hospital of Central South University, Changsha, China; ^3^ Trudeau Institute, Saranac Lake, NY, United States

**Keywords:** *Campylobacter jejuni*, colitis, innate lymphoid cells, interleukin-23, Campylobacteriosis, IL-10

## Abstract

Human pathogen *Campylobacter jejuni* is a significant risk factor for the development of long-term intestinal dysfunction although the cellular and molecular mechanisms remain scantily defined. IL-23 is an emerging therapeutic target for the treatment of inflammatory intestinal diseases, however its role in *C. jejuni*-driven intestinal pathology is not fully understood. IL-10 deficient mice represent a robust model to study the pathogenesis of *C. jejuni* infection because *C. jejuni* infection of mice lacking IL-10 results in symptoms and pathology that resemble human campylobacteriosis. To determine the role of IL-23 in *C. jejuni*-driven intestinal inflammation, we studied the disease pathogenesis in IL-23^-/-^ mice with inhibited IL-10Rα signaling. These mice exhibited reduced intestinal pathology independent from bacterial clearance. Further, levels of IFNγ, IL-17, IL-22, TNF, and IL-6 were reduced and associated with reduced accumulation of neutrophils, monocytes and macrophages in the colon. Flow cytometry analysis revealed reduced production of IL-17 and IFNγ by group 1 and 3 innate lymphoid cells. Thus, our data suggest that IL-23 contributes to intestinal inflammation in *C. jejuni* infected mice by promoting IL-17 and IFNγ production by innate lymphoid cells.

## Introduction

*Campylobacter jejuni* is a foodborne pathogen that is one of the most common causes of human gastroenteritis (1, 2). Although the acute symptoms are usually self-limiting, long-term post infection complications such as Guillain–Barré syndrome, Reiter’s arthritis (3), and irritable bowel syndrome had been described (4–6). Association between *C. jejuni*-driven intestinal pathology and inflammatory bowel disease (IBD) has been also discussed (7, 8). Superimposed *C. jejuni* infection has been frequently identified in ulcerative colitis patients (9, 10). Additionally, an increasing data suggest poorer clinical outcomes in IBD patients following *C. jejuni* infection (9–11). Prior studies established relevant and convenient mouse model of *C. jejuni* infection using mice with IL-10R blockade (2, 12). In this model *C. jejuni* induces intestinal inflammation which mimics pathophysiological features of campylobacteriosis in humans (2, 13). However, very little is known about immune responses that promote intestinal pathology in this model.

In addition to IL-10, IL-23 represents another cytokine controlling the intestinal inflammation. Interleukin-23 (IL-23) is a heterodimeric cytokine of the IL-12 cytokine family, which consists of IL-23p19 subunit and IL-12p40 subunit (14). Activation of IL-23/IL-23R signaling pathway is critical for the development of Th17 responses which are characterized by increased IL-17A, IL-17F and IL-22 production (15, 16). A number of recent reports have shown the crucial role of the IL-23 pathway in murine models of autoimmune and inflammatory diseases (15). IL-23 was identified as the main driver of intestinal inflammation in mouse models of T cell transfer colitis (16–18), anti-CD40 antibody-driven colitis (19), *Clostridium difficile* infection (20, 21), *Helicobacter hepaticus*-induced colitis (18), and *Salmonella enterica* (22). Further evidence that IL-23 is essential for the development of intestinal inflammation not only in mouse models but also in humans comes from clinical studies of IBD patients treated with neutralizing p40 and p19 antibodies. Anti-p40 antibodies were approved for treatment of patients with Crohn`s disease (CD) (14) and anti-p19 antibodies showed promising results for treatment of both CD and ulcerative colitis (UC) patients in initial clinical studies (23, 24). In contrast, a protective role of IL-23 was demonstrated in *Citrobacter rodentium* (25), and *Klebsiella pneumonia* (26) infections by inducing IL-22 production. Therefore, the role of IL-23 can be context-dependent and rely on outcome of complex cytokine signaling in the gut provided by distinct immune cells. The role of IL-23 in *C. jejuni*-induced intestinal pathology remains largely unknown.

Here, we investigated the role of IL-23 in intestinal inflammation caused by *C. jejuni* in an animal model of disease. Our findings suggest that IL-23 promotes pathology by inducing IL-17 and IFNγ production by innate immune cells in early onset of the disease.

## Materials and Methods

### Mice

All studies were conducted in accordance with the University of Texas Health Science Center at San Antonio Animal Care and Use guidelines and biosafety guidelines and approved by Institutional Animal Care and Use Committee (protocol #20170020AR) and Biosafety Committee. Six to twelve-week-old male and female mice were used for experiments. C57BL/6 (wild-type, WT) mice were originally obtained from The Jackson Laboratory (Bar Harbor) and bred at the University of Texas Health Science Center at San Antonio. IL-23^-/-^ and IL-22^-/-^ mice were described previously (27, 28). All mice used in this study were on C57BL/6 background and maintained under specific pathogen free conditions. Age/sex matched controls or littermate controls were used for all experiments.

### IL-10R Blockade

To block IL-10Rα *in vivo*, mice were injected *i.p.* with 350 μg of clone 1B1.3A (BioXCell, West Lebanon, NH) 12–16 h prior to infection and again 1, 4, and 7 days after infection. Isotype mIgG1clone HRPN (BioXCell, West Lebanon, NH) or PBS (Phosphate-buffered saline) was used as control.

### Bacterial Growth and Infection

*Campylobacter jejuni* NCTC 11168 inocula were streaked onto Mueller Hinton (MH) agar and grown at 37 °C under microaerophilic conditions using anaerojars and Oxoid CampyGen sackets (Thermo Scientific) for 48 h. Several colonies were picked from the initial growth and subcultured on MH agar for 24–28 h. Mice were pre-treated with an antibiotic cocktail for seven days in the drinking water as previously described (29). One day after the removal of antibiotics, mice were inoculated with a single dose of 1–5 × 10^9^ CFU of *C. jejuni* or medium by oral gavage. Mice were weighed daily following infection and euthanized if more than 20% of initial body weight was lost.

### Evaluation of Colitis

Ten days after infection, mice were euthanized using CO_2_ inhalation and death was ensured by performing cervical dislocation. The severity of colitis was assessed by hematoxylin and eosin (H&E) staining and colonic mass-to-length ratio. H&E stained sections were evaluated for characteristics of inflammation and images were taken with a Zeiss Axiophot2 microscope (Thornwood, NY). For histopathologic scoring, crypt architecture, mucosal thickening, cellular infiltrates, hemorrhage, goblet cell depletion and tissue damage were evaluated (**Supplementary Table 1**).

### Hydrodynamic IL-23 Plasmid Injection

10 μg of IL-23 p19-p40-expressing plasmid (p-IL-23) (30) or control plasmid (pRK) in 1.7 ml of TransIT-EE Hydrodynamic Delivery Solution (Mirus Bio) were intravenously injected in the tail vein on the second day after *C. jejuni* inoculation.

### Quantitative Analysis of Colon-Associated *C. jejuni*

The distal half of the colon tissue was cut longitudinally and washed three times in sterile PBS. The tissue was weighed, homogenized in MH media, serially diluted and plated on MH agar (Muller-Hinton agar II Agar, BD). Plates were incubated at 42 °C for 48 h before counting.

### Preparation of Colonic Lamina Propria Leukocytes

Lamina propria leukocytes were isolated as previously described (31). Briefly, the cecum and colon were cut open and rinsed twice in PBS to remove fecal material. Colon pieces were incubated in complete medium (RPMI-40 supplemented with 3% FBS, 1 mM penicillin-streptomycin) containing 2 mM EDTA for 20 min at 37 °C with slow rotation (100 r.p.m.) to remove epithelial cells. Remaining tissue pieces were digested in serum-free RPMI-40 containing 200 μg/ml Liberase TM (Roche) and 0.05% DNase I (Sigma) for 40 min at 37 °C with shaking at 100 r.p.m. The digested tissue was passed through a mesh screen, washed with PBS containing 3% FBS and separated by 80/40% Percoll gradient (GE Healthcare).

### Flow Cytometry Analysis

The cells were preincubated for 20 min with anti-CD16/32 (2.4G2, Fc block from BioXCell) and stained for viability with LIVE/DEAD^®^ (Invitrogen, Carlsbad, CA) for 20 min on ice. Cells were surface stained for flow cytometry with the combination of the following antibodies (eBioscience or Biolegend): Anti-CD45 (104), anti-CD3 (145-2C11), anti-Thy1.2 (30-H12), anti-B220 (RA3-6B2), anti-Ter-119 (TER119), anti-Gr-1 (RB6-8C5), anti-CD11c (N418), anti-CD5 (53-7.3), anti-Eomes (Dan11mag), anti-T-bet (4B10), anti-RORγt (Q31-378, BD Pharmingen), anti-IFNγ (XMG1.2), anti-IL-17a (TC11-18H10.1), anti-Ly6G (1A8), anti-CD11b (M1/70), anti-CD64 (X54-5/7.1), anti-CD103 (2E7), anti-MHCII (M5/114.15.2). For intracellular cytokine staining was cells (1 x 10^6^ cells) were restimulated for 4 hours at 37°C with PMA (50 ng/ml) and ionomycin (500 nM) in the presence of brefeldin A (5 μg/ml) (all from Sigma-Aldrich, St. Louis MO) and fixed and permeabilized using fixation and permeabilization solution (eBiosciences). All cells were analyzed on either a FACS Celesta or LSR II (BD Biosciences) and analyzed with FlowJo software (FlowJo LLC).

### Real-Time RT-PCR Analysis

RNA from colon was isolated using E.Z.N.A. Total RNA kit I (Omega Bio-tek). cDNA synthesis and real-time RT-PCR was performed as described previously (32) using Power SYBR Green master mix (Applied Biosystems). Relative mRNA expression of the target gene was determined using the comparative 2^−ΔΔCt^ method. Primers used are listed in **Supplementary Table 2**.

### Statistical Analysis

Statistical analysis was performed using the Mann & Whitney test or two-way analysis of variance (ANOVA) when appropriate. Data are presented as means ± S.E.M. with p < 0.05 being considered significant. All statistical tests were performed in GraphPad Prism 8.0 program. ns, not significant; *p < 0.05, **p < 0.01, ***p < 0.001.

## Results

### IL-23 Drives *C. jejuni*-Induced Intestinal Pathology

**Figure 1:**
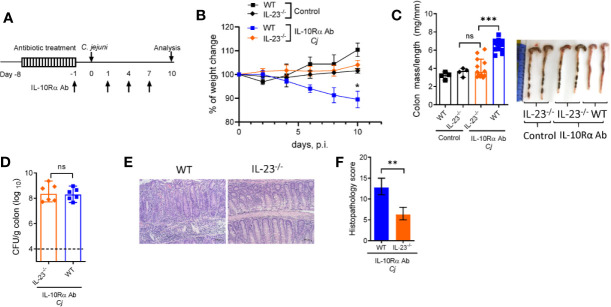
IL-23 is critical for *C jejuni-*induced colitis. Antibiotic pre-treated IL23^-/-^ and WT mice were injected with IL-10Rα Ab on days -1, 1, 4, and 7 and orally inoculated with *Campylobacter jejuni* (*C.j*) or media (Control) **(A)**. Changes in body weight **(B)**, colon mass-to-length measurements and representative photographs **(C)**, bacterial burdens in the colon **(D)** at day 10 post infection. H&E stained colon sections (Scale bars, 100 μm) **(E)** and histopathology scores of colon sections **(F)** from the indicated mice at day ten after infection. Statistical analysis was performed using two-way ANOVA with Bonferroni’s multiple hypothesis correction **(B)** or Mann-Whitney test **(C, D, F)**. *p < 0.05, **p < 0.01, ***p < 0.001; ns, not significant. Data shown are median with 95% of confidence interval, symbols represent individual mice **(C, D)**. Dotted horizontal lines represent the limit of detection **(D)**. Data represents two-four pooled experiments (n = 4–12 per group).

IL-23 is known to participate in both protective and pathogenic responses in the gut (17, 18, 33). To define the role of IL-23 in pathogenesis of *C. jejuni*-induced intestinal inflammation, we used a previously described animal model of Campylobacteriosis using mice with IL-10R blockade which mimics pathophysiological features of human disease (2, 34). To facilitate *C. jejuni* colonization, IL-23^-/-^ and WT control mice were pre-treated with broad spectrum antibiotics, as described (34) and orally infected with *C. jejuni* (**Figure 1A**). *C. jejuni* infection induced severe intestinal inflammation in aIL-10Rα antibody-treated WT mice, as demonstrated by significant weight loss (**Figure 1B**), colon thickening and increased colonic mass-to-length ratio (**Figure 1C**). Histological analysis revealed inflammatory lesions in both cecum and colon of infected WT mice (**Figures 1E, F**). In stark contrast, IL-23^-/-^ mice did not show significant body weight loss and colon thickening (**Figures 1B, C**). Although the IL-23-deficiency did not affect *C. jejuni* colonization of the intestine (**Figure 1D**), the histological evaluation of colons revealed reduced intestinal inflammation with minor immune cell infiltration, epithelial damage and crypt hyperplasia compared to WT mice (**Figures 1E, F**). Accordingly, although WT and IL-23^-/-^ mice without aIL-10Rα treatment displayed similar *C. jejuni* colonization (**Supplementary Figures 1B, C**), intestinal pathology was reduced in IL-23 deficient mice (**Supplementray Figure 1A**). Together, these observations indicate that IL-23 drives pathology in *C. jejuni* infected mice.

### IL-23 Drives Accumulation of Myeloid Cell Populations in the Colon of *C. jejuni*-Infected Mice

**Figure 2:**
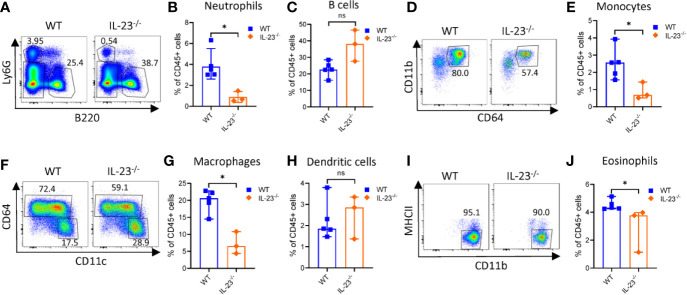
Analysis of intestinal myeloid populations in IL23^-/-^ mice during *C. jejuni*-induced colitis. IL23^-/-^ and WT mice were treated as on **Figure 1A**. Ten days later, cells were purified from colon lamina propria. Live CD45^+^ cells were analyzed for expression of Ly6G, MHCII, Ly6C, CD11c, CD11b, and CD64. **(A)** Representative plots of neutrophils (Ly6G^+^) and B cells (B220^+^). **(B)** Frequencies of neutrophils and **(C)** B cells. **(D, E)** Representative plots and frequencies of monocytes (Ly6G^-^MHCII^-^CD64^+^CD11b^+^). **(F–H)** Representative plots and frequencies of macrophages (CD64^+^MHCII^+^CD11b^+^) and dendritic cells (CD64^-^CD11c^+^MHCII^+^). **(I, J)** Representative plots and frequencies of eosinophils (CD45^+^Ly6G^-^CD11b^+^CD11c^-^ MHCII^-^). Statistical analysis was performed using Mann-Whitney test. *p < 0.05; ns, not significant. Data shown are median with 95% of confidence interval, symbols represent individual mice. Data represents one experiment (n = 3–5 per group).

*Campylobacter jejuni*-induced intestinal inflammation is associated with infiltration of myeloid cells such as neutrophils, monocytes, macrophages and dendritic cells (DCs) to the large intestine (29, 35). Given that *C. jejuni* infected IL-23^-/-^ mice did not develop significant intestinal pathology, we next asked if the accumulation of myeloid populations were impaired in these mice. Evaluation of colonic myeloid cell populations demonstrated fewer frequency of neutrophils, eosinophils (identified by a negative gating, **Supplementary Figure 2A**), macrophages and monocytes among CD45^+^ immune cells compared to WT mice on day 10 after infection (**Figures 2A, B, D–G, I, J**). By contrast, there was no significant difference in DCs and B cells between IL-23^-/-^ and WT mice (**Figures 2C, H**). These results demonstrate that IL-23-driven response is essential for the accumulation of neutrophils, eosinophils, macrophages and monocytes in the large intestine during *C. jejuni* induced colitis.

**Figure 3:**
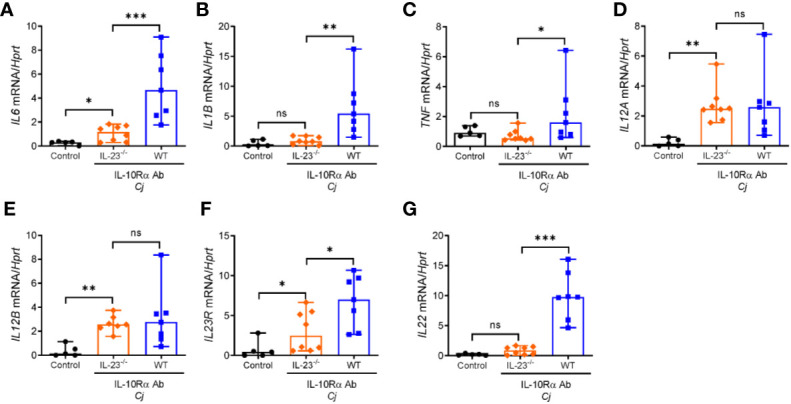
Abrogated expression of proinflammatory cytokines in the colon of *C. jejuni*-infected IL-23 deficient mice. IL23^-/-^ and WT mice were treated as on **Figure 1A**. mRNA expression of cytokines in the colon were measured by real-time PCR on day 10 post infection. **(A–G)** Antibiotic pretreated mock-infected WT mice were used as a control. Statistical analysis was performed using Mann-Whitney test. *p < 0.05, **p < 0.01, ***p < 0.001; ns, not significant. Data shown are median with 95% of confidence interval, symbols represent individual mice. Data represents two experiment (n = 5–8 per group).

Innate immune cells such as neutrophils, macrophages and dendritic cells upon activation can produce multiple cytokines that can activate and facilitate recruitment of circulating lymphocytes and inflammatory myeloid cells to the sites of inflammation. Previously, it was shown that expression of proinflammatory cytokines such as TNF, IL-1β, IL-12, and IL-6 was increased in the colon of *C. jejuni*-infected IL-10^-/-^ mice (29, 35, 36). To further examine the role of IL-23 in *C. jejuni* induced inflammatory response, we assessed the expression of these cytokines in the colons of infected mice at day 10 post infection. As shown in **Figure 3**, the mRNA levels of IL-12p35 and IL-12p40 subunits were unchanged in IL-23^-/-^ mice compared to WT mice, indicating that IL-23 deficiency is not associated with impaired expression of IL-12. In contrast, IL-6, IL-1β, IL-23R, and TNF were downregulated in IL-23^-/-^ mice (**Figures 3A–C, F**). As IL-23 is one of the major cytokines regulating IL-22 expression in inflammatory conditions (30, 37), we next measured IL-22 levels. Indeed, IL-22 expression was significantly reduced in IL-23^-/-^ mice after *C. jejuni* infection (**Figure 3G**).

These data indicate that IL-23 regulates accumulation of myeloid cells in the gut during *C. jejuni*-induced colitis. Moreover, IL-23 deficiency diminishes expression of proinflammatory cytokines, most likely due to reduced infiltration of myeloid cells to the intestine.

### IL-22 Is Dispensable for *C. jejuni-*Induced Intestinal Inflammation

**Figure 4:**
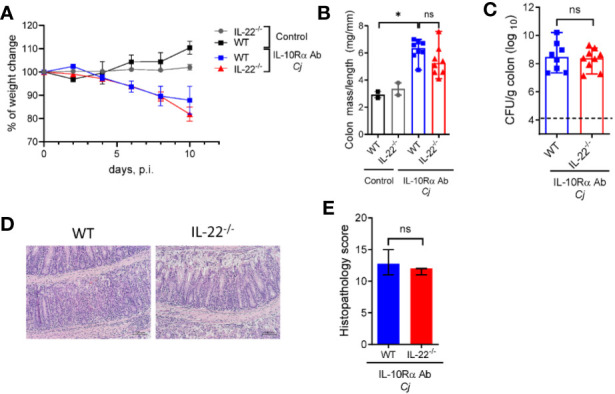
IL-22 is dispensable for *C. jejuni-*induced colitis. IL-22^-/-^ and WT mice were treated as on **Figure 1A**. Changes in body weight **(A)**, colon mass-to-length ratio **(B)** and bacterial burdens in the gut **(C)** were determined 10 days after infection. **(D)** H&E stained colon sections (Scale bars, 100 μm) and **(E)** histopathology scores of colon sections from the indicated mice at day ten after infection. Statistical analysis was performed using two-way ANOVA with Bonferroni’s multiple hypothesis correction **(B)** or Mann-Whitney test **(B, C, E)**. *p < 0.05; ns, not significant Data shown are median with 95% of confidence interval, symbols represent individual mice. **(B–D)**. Dotted horizontal lines represent the limit of detection **(C)**. Data represents two pooled experiments (n = 2–8 per group).

Numeral studies have demonstrated that IL-22 can regulate intestinal inflammation by promoting epithelial cell repair and production of anti-microbial peptides (38). IL-22 can also promote intestinal pathology in several models of intestinal inflammation driven by CD40 (19), *Toxoplasma gondii* (39) and in a model of spontaneous colitis caused by IL-10 deficiency (40). IL-23 promotes IL-22 production mainly by T cells and ILCs during mucosal bacterial infection (41, 42). We found that colonic mRNA expression of IL-22 was significantly increased in WT mice after *C. jejuni* infection (**Figure 3G**), consistent with previous studies (35, 43). To further examine the role of IL-22 in *C. jejuni*-induced intestinal pathology, we infected aIL-10Rα-blocked IL-22^-/-^ mice according to the same protocol (**Figure 1A**) and assessed the development of disease. Surprisingly, there was no significant difference in body weight change, colonic mass-to-length ratio and bacterial burdens in IL-22^-/-^ mice compared to WT mice (**Figures 4A–C**). Similarly, no significant difference in histopathological score was observed between infected WT and IL-22^-/-^ mice (**Figures 4D, E**). Together these results indicate that IL-22 is not essential for *C. jejuni-*induced intestinal pathology in this model.

### IL-23 Contributes to IL-17 and IFNγ Production by ILCs

**Figure 5:**
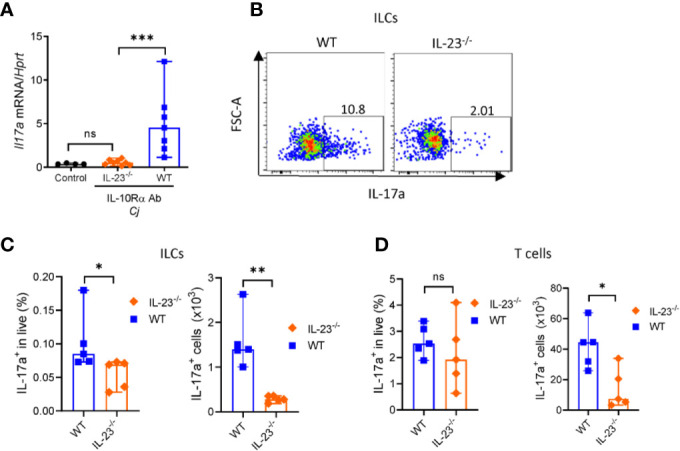
IL-23 is required for IL-17 production during *C. jejuni* induced colitis. IL23^-/-^ and WT mice were treated as on **Figure 1A**. On day 10 colonic tissue was harvested, and **(A)** expression of IL-17a was measured by real-time PCR. Antibiotic pretreated mock-infected mice WT mice were used as a control. **(B–D)** On day 10 post-infection cells were isolated from the large intestine of IL23^-/-^ and WT mice and stimulated for 4 h with P/I, and BFA before analysis for IL-17a by flow cytometry. **(B, C)** Representative plots of IL-17 production by ILCs (CD3^-^Lin^-^Thy1.2^+^Eomes^-^) and frequencies and absolute numbers of IL-17a^+^ ILCs. **(D)** Frequencies and absolute numbers of IL-17a^+^ T cells (CD3^+^). Statistical analysis was performed using Mann-Whitney test. *p < 0.05, **p < 0.01, ***p < 0.001; ns, not significant. Data shown are median with 95% of confidence interval, symbols represent individual mice. Data represents two experiment (n = 2–3 per group).

*C. jejuni*-induced colitis in IL-10^-/-^ mice is characterized by mixed Th1 and Th17 responses (35, 43). Recently it has been shown that both IFNγ and IL-17, signature cytokines of Th1 and Th17 responses, respectively, contribute to *C. jejuni*-induced pathology (35, 43). Depletion of either IFNγ or IL-17 or both during *C. jejuni* infection significantly decreased inflammation and pathology (35, 44). We showed that colonic IL-17 expression was decreased in IL-23^-/-^ mice compared to WT mice (**Figure 5A**). IL-23 can drive IL-17 production by both CD4^+^ T cells and innate lymphoid cells (ILCs) (45).

To determine which of these cells contribute to IL-23-dependent IL-17 production in *C. jejuni* infection, we isolated cells from colonic lamina propria of IL-23^-/-^ mice. Cells were stimulated with PMA and ionomycin in the presence of Brefeldin A and IL-17 production by T cells and ILCs was analyzed by flow cytometry (**Supplementary Figures 2B, C**). Our analysis revealed that the total number and frequency of IL-17 producing ILCs were reduced in IL-23^-/-^ mice (**Figures 5B, C**), however only total number of IL-17^+^ T cells was significantly reduced (**Figure 5D**). These data suggest that both ILCs and T cells contribute to IL-23 dependent IL-17-production during *C. jejuni* infection.

**Figure 6:**
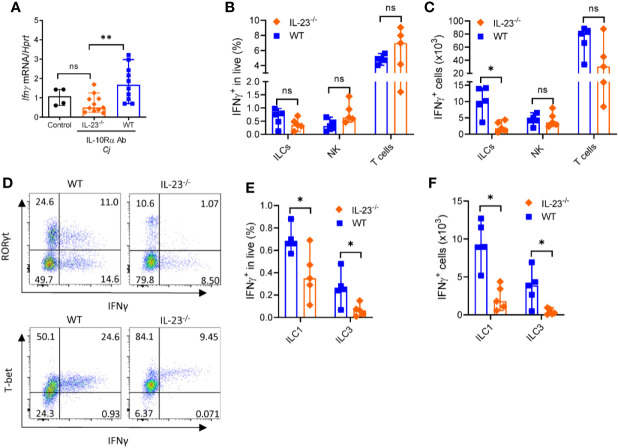
IL-23 is required for IFNγ production during *C. jejuni*-induced colitis. IL23^-/-^ and WT mice were treated as on **Figure 1A** and analyzed on day 10. **(A)** Expression of IFNγ was measured by real-time PCR. Antibiotic pretreated mock-infected mice WT mice were used as a control. **(B–F)** Cells were isolated from the large intestine of IL23^-/-^ and WT mice and stimulated for 4 h with PMA/ionomycin, and brefeldin A. **(B, C)** Frequencies and absolute numbers of IFNγ^+^ ILCs (CD3^-^Lin^-^Thy1.2^+^Eomes^-^), NK cells (CD3^-^T-bet^+^Eomes^+^) and T cells (CD3^+^). **(D)** Representative plots of ILC1 and ILC3 IFNγ -producing cells. **(E, F)** Frequencies and absolute numbers of IFNγ^+^ ILC1s (CD3^-^Lin^-^Thy1.2^+^Eomes^-^ T-bet^+^) and ILC3s (CD3^-^Lin^-^Thy1.2^+^Eomes^-^ RORγt^+^). Statistical analysis was performed using Mann-Whitney test. Statistical significance indicated by *p < 0.05, **p < 0.01; ns, not significant. Data shown are median with 95% of confidence interval, symbols represent individual mice. Data represents two experiment (n = 2–3 per group).

The major producers of IFNγ during *C. jejuni* infection are T cells, ILCs and NK cells (35, 44). Our data suggest that IL-23^-/-^ mice display reduced IFNγ expression compared to WT mice (**Figure 6A**). We next asked if the production of IFNγ by these cells was affected by IL-23 deficiency during *C. jejuni* infection. We found a marked reduction of IFNγ-producing ILCs but not NK or T cells in the colon of IL-23^-/-^ mice on day 10 after *C. jejuni* infection (**Figures 6B, C**). aIL-10Rα antibody treatment in uninfected mice did not affect the number of IFNγ-producing ILCs and T cells in the colon (**Supplementary Figures 3A, B**), in agreement with published report (44). In contrast to colon, we did not find difference in IFNγ production by ILCs or T cells in MLN between WT and IL-23^-/-^ infected mice (**Supplementary Figures 3C–F**). These data suggest that IL-23 contributes to IFNγ production in the colon during *C. jejuni* infection. To further define if IL-23 regulates IFNγ-production in another gut bacterial infection model, we infected IL-23^-/-^ mice with *Citrobacter rodentium* and analyzed IFNγ-expression on day 5 p.i. We did not detect any difference in IFNγ-expression between IL-23^-/-^ and control mice (data not shown), in agreement with protective role of IL-23 in this infection (25, 46).

Previous studies demonstrated that IFNγ can be produced by group 1 ILCs characterized by expression of signature transcription factor T-bet and by group 3 ILCs characterized by RORγt expression (47, 48). As total cell number of IFNγ^+^ ILCs was selectively reduced in IL-23 deficient mice, we next asked which subtypes of ILCs were affected. As shown in **Figures 6D–F**, both frequency and total cell number of IFNγ-producing ILC1s and ILC3s were significantly reduced in IL-23^-/-^ mice after *C. jejuni* infection, demonstrating that IL-23 is important to promote IFNγ-production by these ILCs during *C. jejuni*-induced colitis.

To further define if IL-23 contributes to *C. jejuni*-induced intestinal pathology, we used hydrodynamic injection of a plasmid encoding both p19 and p40 IL-23 subunits (p-IL-23) (30). We found that p-IL-23 treated mice lost significantly more weight and displayed increased levels of IFNγ, IL-17 and IL-22 in the colon (**Supplementary Figure 4**). These data further support the pathogenic role of IL-23 in *C. jejuni*-induced intestinal pathology.

Collectively, these data indicate that IL-23 signaling contributes to *C. jejuni*-induced intestinal inflammation by driving IL-17 and IFNγ responses by T cells and ILCs.

## Discussion

Accumulating evidence suggests that *C. jejuni-*induced intestinal pathology is driven by excessive activation of host immune responses (49, 50). At the same time, immune responses are required for pathogen containment and elimination (51). Therefore, uncoupling protective host defense mechanisms from the immunopathology induced by these mechanisms is critical for therapeutic management of *C. jejuni*-driven intestinal disease.

The goal of this study was to define the role of IL-23 in the pathogenesis of *C. jejuni*-induced colitis. Mouse model with blocked IL-10R signaling provides a robust platform to study immune mechanisms of intestinal disease pathogenesis as *C. jejuni* colonization in WT mice does not result in body weight loss and severe intestinal pathology (35). Our results suggest that IL-23 promotes pathogenesis of *C. jejuni*-induced colitis by recruiting myeloid cell populations in the colon and enhancing IL-17 and IFNγ responses by group 1 and 3 innate lymphoid cells.

Although the requirement for IL-23 to promote intestinal pathology is consistent with previous findings in other models (18, 20, 21), our study highlights a novel role of IL-23 in driving pathogenic immune responses to *C. jejuni* infection. The cellular events leading to the development of *C. jejuni*-induced intestinal disease are associated with activation and massive infiltration of innate immune cells such as macrophages, neutrophils, dendritic cells and eosinophils to the colon (29, 34). IL-23 is known inducer of IL-17 which activates endothelial and myeloid cells for expression of proinflammatory cytokines including IL-1β, TNF and IL-6 (14, 27, 52). Our data demonstrate that IL-23 deficient mice display downregulated expression of proinflammatory cytokines following *C. jejuni* infection, including IL-17. Accordingly, our flow cytometry analysis revealed reduced production of IL-17 by ILCs and T cells in IL-23 deficient mice. Pathogenic role of IL-17 in *C. jejuni*-induced colitis was previously implicated as IL-17 blocking antibody ameliorated intestinal inflammation in IL-10^-/-^ mice (35). Our results suggest that IL-23-dependent IL-17 production may impact immune cell accumulation and their activation in the intestine during *C. jejuni*-induced colitis. In fact, we found that IL-23 deficient mice exhibited reduced accumulation of neutrophils, monocytes, macrophages and eosinophils in colon compared to WT mice following *C. jejuni* infection. These results are consistent with a previous report of *C. jejuni* infection in infant IL-23^-/-^ mice with intact IL-10R signaling (53), indicating reduced accumulation of neutrophils in the colon during *C. jejuni* infection by immunohistochemistry. Additionally, our flow cytometry analysis revealed reduced numbers of eosinophils, monocytes and macrophages in IL-10Rα-blocked IL-23^-/-^ mice, indicating the role of IL-23 in controlling these cell populations in this model. It is also possible that neutrophils can be regulated directly by IL-23 signaling pathway, whereas the reduction of other immune cells is affected by both IL-23- and IL-10R- deficient environments.

IL-23 is the major regulator of IL-22 secretion upon inflammation in the gut (30, 42). The role of IL-22 during inflammation is controversial and depends on the context, it can either be pathogenic or protective (42, 54). Recent study indicated a pathogenic role of IL-22 in the model of spontaneous colitis in IL-10^-/-^ mice (40), whereas other studies demonstrated a protective role for IL-22 in host defense against *C. difficile* and *C. rodentium* mucosal pathogens (25, 55). We found that IL-22^-/-^ mice with blocked IL-10Rα displayed similar intestinal pathology and bacterial titers compared to WT mice. Interestingly, previous study showed increased *C. jejuni* titers in colon of IL-22^-/-^ mice with intact IL-10R signaling (53) indicating a protective role of IL-22 in controlling bacterial colonization. The absence of IL-22 leads to altered microbiota in the colon which can affect the degree of colonization and disease course (56). Moreover, it should be noted that *C. jejuni* disease has a large spectrum of outcomes due to high genetic diversity of different strains (57). In our model we used NCTC 11168 that is less invasive compared to ATCC 43431 strain which was used in another study (57). Thus, our results suggest that IL-22 is dispensable for intestinal pathology in our model.

IFNγ is a proinflammatory cytokine that is induced in colon during *C. jejuni* infection. Neutralizing of IFNγ prevents the development of severe *C. jejuni*-mediated colitis in IL-10^-/-^ mice (35). In our experiments, the major producers of IFNγ were T cells, NK cells and ILCs, in agreement with previous reports (35). Surprisingly, we found that genetic ablation of IL-23 resulted in reduction of IFNγ-producing ILCs but not T cells during *C. jejuni* infection suggesting that ILCs can contribute to colitis development by IFNγ production during early stages of infection. Additionally, our recent data indicated that inactivation of T-bet in NKp46^+^ ILCs reduced intestinal pathology highlighting the pathogenic role of IFNγ^+^ ILCs during early stages of infection (44). A previous study showed that CD4^+^ T cells in IL-10^-/-^ mice produced similar IFNγ levels compared to CD4^+^ T cells in IL-23^-/-^IL-10^-/-^ mice in spontaneous model of colitis (52), indicating normal Th1 response in IL-23- and IL-10-deficient environment. In line with our results, recent study highlighted distinct role of IFNγ produced by T cells versus ILCs in promoting intestinal inflammation in different experimental animal models (58). Additionally, IL-23-responsive ILCs can contribute to chronic intestinal inflammation in *H. hepaticus* innate colitis model through production of inflammatory cytokines, including IFNγ (59). Based on the data presented here, we hypothesize that IL-23 amplifies local expression of proinflammatory cytokines (IL-1, IL-6, and TNF), which in turn can stimulate the release of additional proinflammatory mediators by other cell populations including T cells and ILCs. Thus, our results suggest that IL-23 can promote intestinal pathology *via* regulation of IFNγ production by ILCs.

ILCs are located at mucosal tissues and can quickly respond to pathogen invasion by local production of signature cytokines. ILC1 and ILC3 previously have been reported to express T-bet and are capable to produce IFNγ after stimulation (60, 61). Recent studies in animal models of IBD showed that ILCs contribute to inflammatory response and intestinal pathology (59, 62). ILC1 and ILC3 coordinate early host response through secretion of IFNγ and IL-22 in the acute phase of *C. difficile* infection (63). Several studies showed that IL-23 can directly regulate activation of ILC3s during inflammation (59). Upon activation ILC3 can release not only IFNγ but also IL-17. Moreover, the imbalance between IL-23 and IL-12 can induce the transition between ILC3 and ILC1 subsets (61). Thus, the dysregulation of ILCs immune response has been involved in development of IBD. Our data indicate that both IFNγ- and IL-17-producing ILC1 and ILC3 subsets were reduced in IL-23-deficient mice. Consistently, increased number of IFNγ^+^ ILCs in colon of IL-10^-/-^ mice at day 4 after *C. jejuni* infection was reported by previous study (35). Therefore, the reduction of both IL-17 and IFNγ-production by ILCs in early onset of disease can lead to impaired development of inflammation in IL-23^-/-^ mice during *C. jejuni* infection. While our data demonstrate that both IFNγ and IL-17 production was reduced by ILCs in IL-23-deficient mice, we also do not exclude the role of T cells in *C. jejuni*-induced intestinal pathology especially during later stages of infection when adaptive immune response takes place. Further studies will be necessary to define whether IL-23 can promote *C. jejuni*-induced intestinal pathology *via* direct stimulation of innate immune cells, particularly ILCs, or indirect mechanism involving other immune cells during early stages of infection.

Accumulating evidence suggests that *C. jejuni* infection can contribute to pathogenesis of IBD, however *C. jejuni*-mediated pathogenic immune mechanisms remain poorly understood (7, 8). Genome-wide association studies revealed SNP in *IL23R* and *IL10* genes, providing evidence for the important role of the IL-23 and IL-10 signaling pathways in the pathogenesis of IBD (64–66). T cells, monocytes, macrophages and DCs are major sources of IL-10 (67). In the context of impaired IL-10 signaling, monocytes that respond to bacteria can express IL-23 as a result of acquired IL-10 signaling resistance (68). Studies on samples from patients with IBD showed that inflammatory monocytes in a state of IL-10 non-responsiveness produce high levels of IL-23 (68). Although those patients express high levels of IL-10, the inflammation is still present due to dysregulated IL-10 responses and intestinal antimicrobial immunity (69, 70). Therefore, the investigation of IL-23 signaling pathway in models of bacterial-induced intestinal inflammation with blocked IL-10R signaling can provide insights to understanding the immune regulations in the gut of IBD patients with dysregulated IL-10 responses.

In summary, we described a crucial role of IL-23 in pathogenesis of *C. jejuni*-induced intestinal disease. Our results highlight the role of IL-23 signaling pathway to modulate both IL-17 and IFNγ production by ILCs in response to *C. jejuni* infection.

## Data Availability Statement

The raw data supporting the conclusions of this article will be made available by the authors, without undue reservation.

## Ethic Statement

The animal study was reviewed and approved by Institutional Animal Care and Use Committee.

## Author Contributions

Study concept and design: EK and AT. Designed and performed experiments, analyzed data, wrote manuscript: XJ, AK, EK, and AT. Performed experiments: WM and SS. All authors contributed to the article and approved the submitted version.

## Funding

This research was supported by grant from NIH (AI135574 and AI111000). WM was supported by USDA NIFA (2014-67012-22276). AT was supported by the Crohn’s and Colitis Foundation (SRA#294083), the Max and Minnie Tomerlin Voelcker Fund, and the William and Ella Owens Medical Research Foundation. Data was generated in the Flow Cytometry Shared Resource Facility which is supported by UT Health San Antonio, NIH-NCI P30 CA054174-20 (CTRC at UTHSCSA) and UL1 TR001120 (CTSA grant).

## Conflict of Interest

The authors declare that the research was conducted in the absence of any commercial or financial relationships that could be construed as a potential conflict of interest.

## Supplementary Material

Supplementary Figure 1 IL-23 is not required for *C. jejuni* colonization. Antibiotic pre-treated IL23^-/-^ and WT mice were orally inoculated with *Campylobacter jejuni* (*Cj*) or media, and treated with aIL-10Rα Ab or isotype control. **(A)** colon mass-to-length. **(B)** bacterial titers in colon. **(C)** bacterial titers in spleen and mesenteric lymph node (MLN). Statistical analysis was performed using Mann-Whitney test. *p < 0.05, ***p < 0.001; ns, not significant. Data shown are median with 95% of confidence interval, symbols represent individual mice. Data represents two-four pooled experiments (n = 3–13 per group).

Supplementary Figure 2 Gating strategies for intestinal myeloid cells and ILCs. **(A)** Gating strategy for myeloid cells from lamina propria. **(B)** Gating strategy for ILCs that were identified as live CD3^-^Lin^-^Thy1.2^+^ cells. Lineage markers were CD11c, B220, Gr-1, and Ter-119. **(C)** Gating strategy for NK cells. Gating was performed on lymphocytes and doublets were excluded. NK cells were identified as live^+^CD3^-^T-bet^+^Eomes^+^ cells

Supplementary Figure 3
*C. jejuni* infected mice exhibit increased levels of IFNγ-producing T cells and ILCs. **(A, B)** Antibiotic pre-treated WT mice were orally inoculated with *C.jejuni* and treated with aIL-10Rα Ab or isotype control. Colonic IFNγ T cells (CD3^-^Lin^-^Thy1.2^+^Eomes^-^) and ILCs (CD3^-^Lin^-^Thy1.2^+^Eomes^-^) were analyzed by flow cytometry at day 10. **(C–G)** Antibiotic pre-treated IL23^-/-^ and WT mice were orally inoculated with *C. jejuni*. Cells were isolated from MLN and IFNγ expression analyzed by flow cytometry. Statistical analysis was performed using Mann-Whitney test. *p < 0.05; ns, not significant. Data shown are median with 95% of confidence interval, symbols represent individual mice. Data represents two experiments (n = 3–5 per group).

Supplementary Figure 4 IL-23 exacerbates Campylobacter *jejuni-*induced colitis. Antibiotic pre-treated IL-10^-/-^ mice were orally inoculated with *C. jejuni* and analyzed at day 6. **(A)** Schematic of the experiment. **(B)** Changes in body weight. **(C–E)** Colon mass-to-length with representative photographs and bacterial burden in colon. **(F–H)**. mRNA expression in colon by real-time PCR. Statistical analysis: two-way ANOVA with Bonferroni’s multiple hypothesis correction **(B)** or Mann-Whitney test **(C–H)**. *p < 0.05, **p < 0.01, ***p < 0.001; ns, not significant. Data shown are median with 95% of confidence interval. Data represents two pooled experiments (n = 4 per group).

Supplementary Table 1 Histological scores evaluation.

Supplementary Table 2 Primers for quantitative PCR.
