# Supplementary figures and images for: IL-23 Contributes to Campylobacter jejuni-Induced Intestinal Pathology via Promoting IL-17 and IFNγ Responses by Innate Lymphoid Cells
Source: Front Immunol. 2021 Jan 6;11:579615. doi: 10.3389/fimmu.2020.579615 (PMC7815532; doi:10.3389/fimmu.2020.579615)

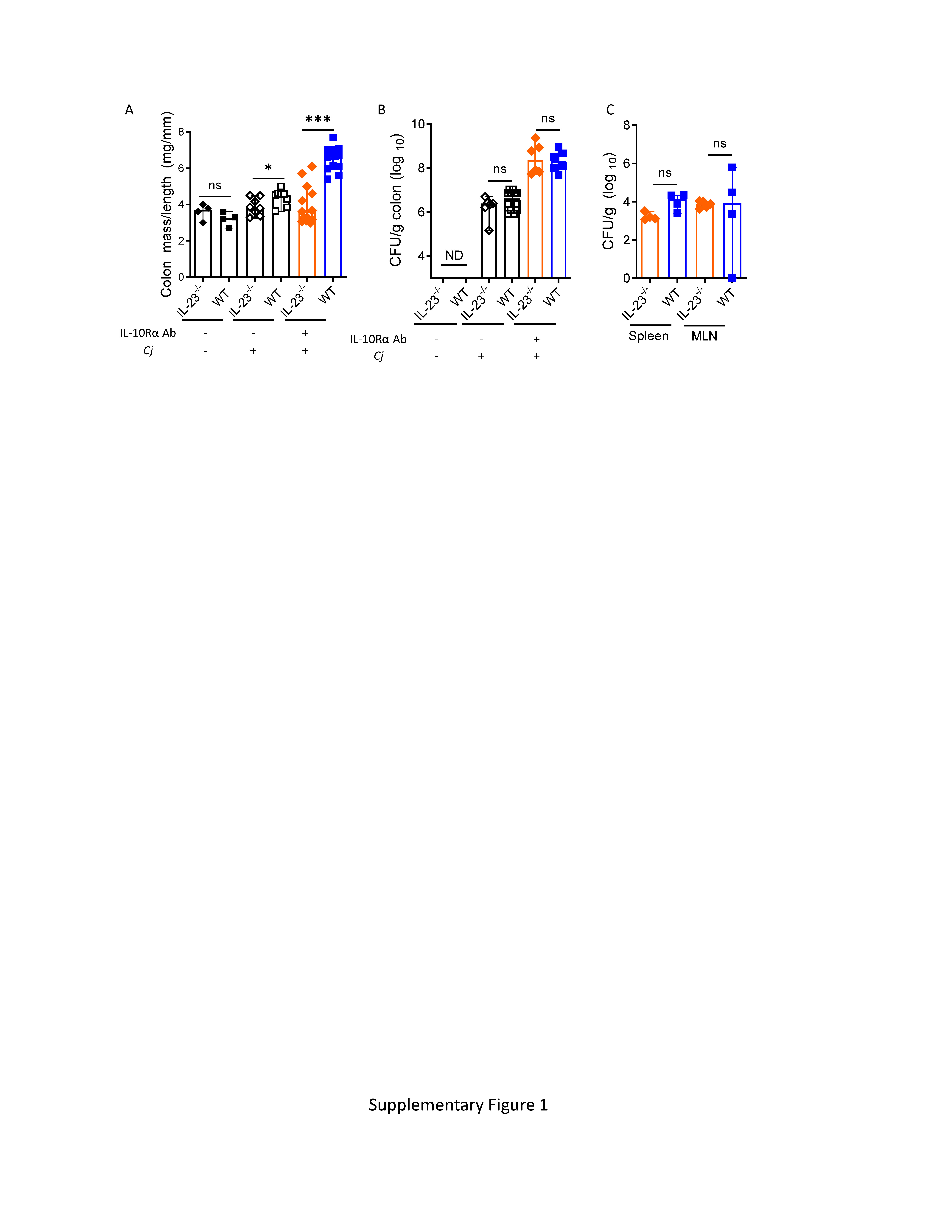

Supplement: Supplementary Figure 1 — IL-23 is not required for C. jejuni colonization. Antibiotic pre-treated IL23-/- and WT mice were orally inoculated with Campylobacter jejuni (Cj) or media, and treated with aIL-10Rα Ab or isotype control. (A) colon mass-to-length. (B) bacterial titers in colon. (C) bacterial titers in spleen and mesenteric lymph node (MLN). Statistical analysis was performed using Mann-Whitney test. *p < 0.05, ***p < 0.001; ns, not significant. Data shown are median with 95% of confidence interval, symbols represent individual mice. Data represents two-four pooled experiments (n = 3–13 per group). [file Image_1.jpeg]

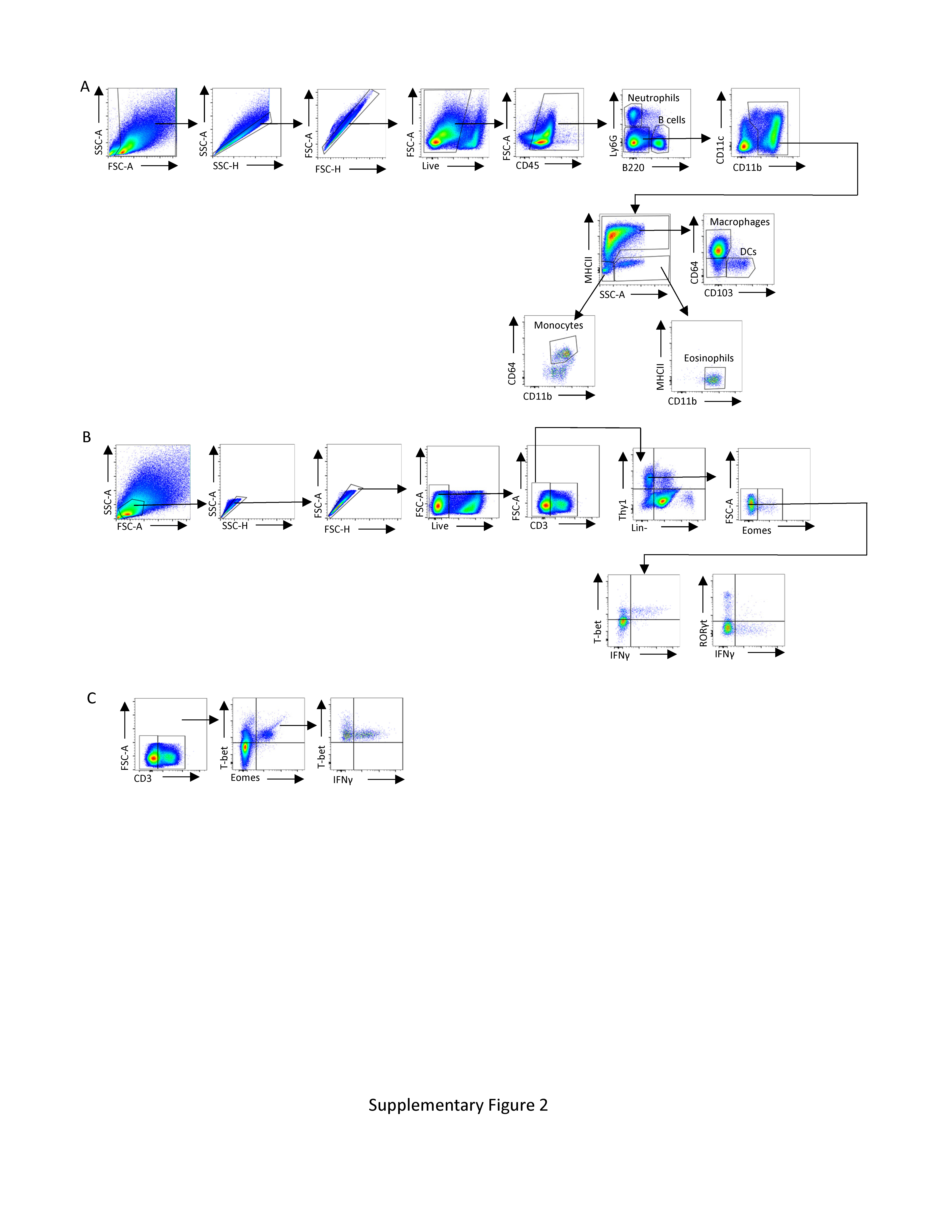

Supplement: Supplementary Figure 2 — Gating strategies for intestinal myeloid cells and ILCs. (A) Gating strategy for myeloid cells from lamina propria. (B) Gating strategy for ILCs that were identified as live CD3-Lin-Thy1.2+ cells. Lineage markers were CD11c, B220, Gr-1, and Ter-119. (C) Gating strategy for NK cells. Gating was performed on lymphocytes and doublets were excluded. NK cells were identified as live+CD3-T-bet+Eomes+ cells [file Image_2.jpeg]

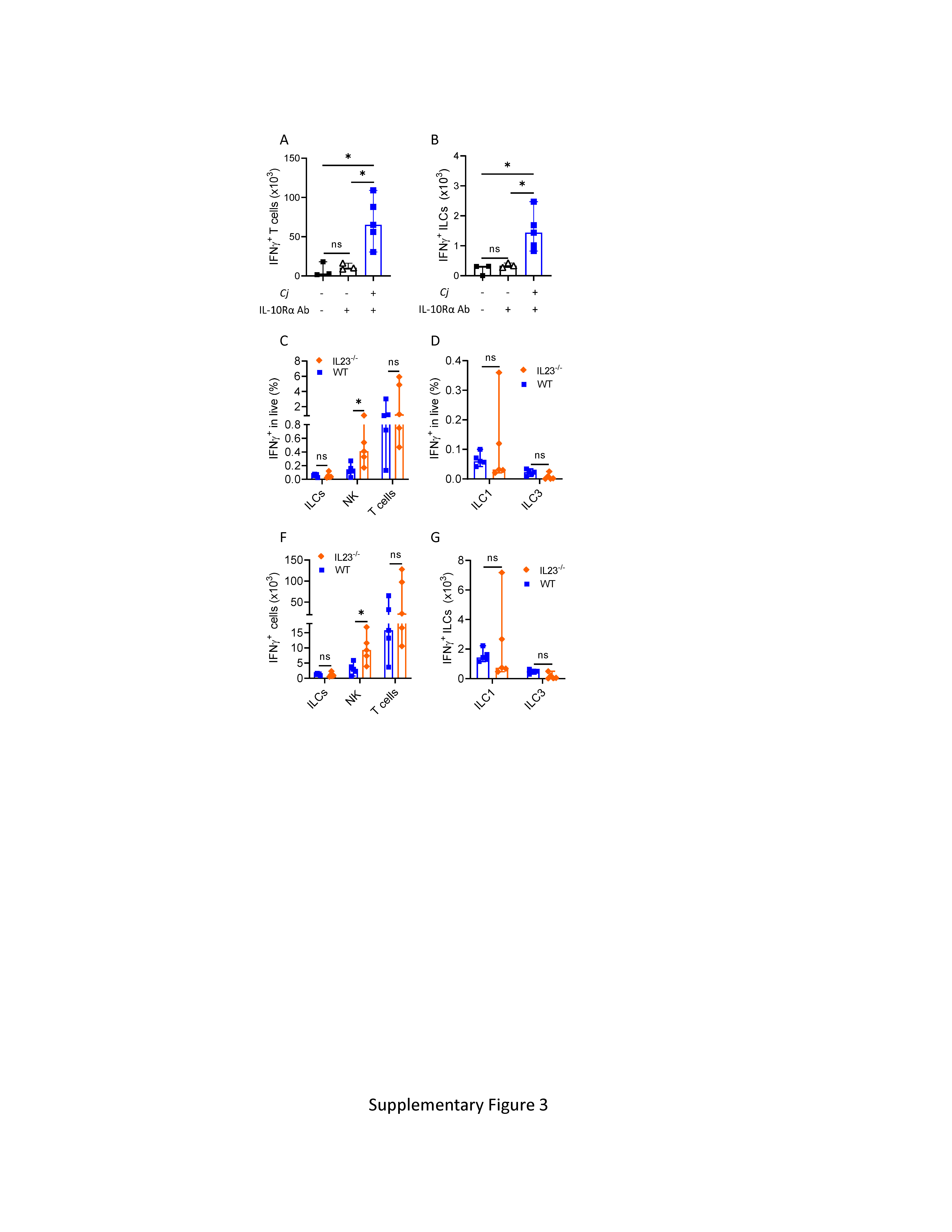

Supplement: Supplementary Figure 3 — C. jejuni infected mice exhibit increased levels of IFNγ-producing T cells and ILCs. (A, B) Antibiotic pre-treated WT mice were orally inoculated with C.jejuni and treated with aIL-10Rα Ab or isotype control. Colonic IFNγ T cells (CD3-Lin-Thy1.2+Eomes-) and ILCs (CD3-Lin-Thy1.2+Eomes-) were analyzed by flow cytometry at day 10. (C–G) Antibiotic pre-treated IL23-/- and WT mice were orally inoculated with C. jejuni. Cells were isolated from MLN and IFNγ expression analyzed by flow cytometry. Statistical analysis was performed using Mann-Whitney test. *p < 0.05; ns, not significant. Data shown are median with 95% of confidence interval, symbols represent individual mice. Data represents two experiments (n = 3–5 per group). [file Image_3.jpeg]

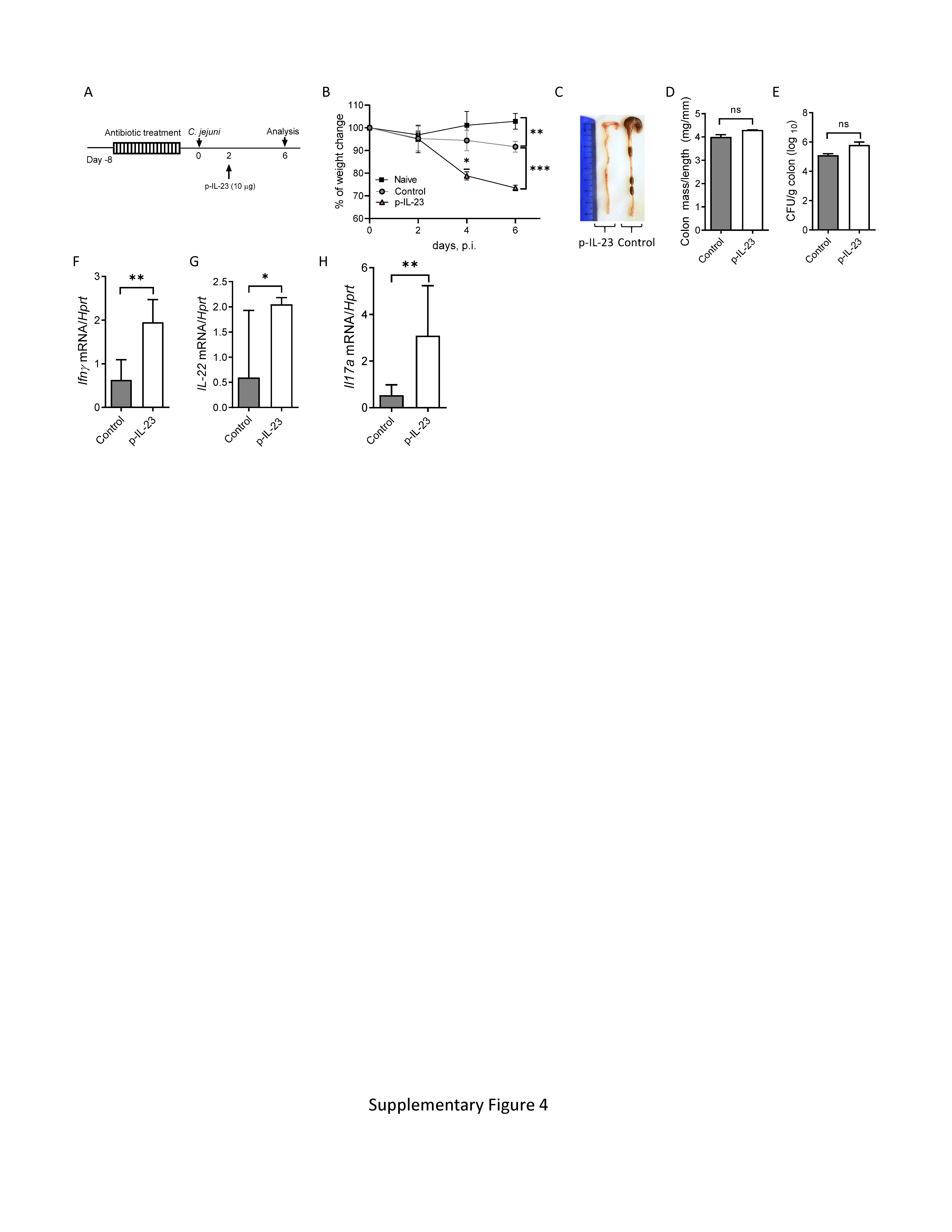

Supplement: Supplementary Figure 4 — IL-23 exacerbates Campylobacter jejuni-induced colitis. Antibiotic pre-treated IL-10-/- mice were orally inoculated with C. jejuni and analyzed at day 6. (A) Schematic of the experiment. (B) Changes in body weight. (C–E) Colon mass-to-length with representative photographs and bacterial burden in colon. (F–H). mRNA expression in colon by real-time PCR. Statistical analysis: two-way ANOVA with Bonferroni’s multiple hypothesis correction (B) or Mann-Whitney test (C–H). *p < 0.05, **p < 0.01, ***p < 0.001; ns, not significant. Data shown are median with 95% of confidence interval. Data represents two pooled experiments (n = 4 per group). [file Image_4.jpeg]
