# Supplementary material for: IL-23 Contributes to Campylobacter jejuni-Induced Intestinal Pathology via Promoting IL-17 and IFNγ Responses by Innate Lymphoid Cells
Source: Front Immunol. 2021 Jan 6;11:579615. doi: 10.3389/fimmu.2020.579615 (PMC7815532; doi:10.3389/fimmu.2020.579615)
Supplement: Supplementary Table 1 — Histological scores evaluation. [file Table_1.pdf]

Supplementary Table 1. Histological scores evaluation

Inflammatory cell infiltration		Epithelial changes	Crypt hyperplasia	Submucosal edema
Extent	severity			
0=none	0=none	0=none	0=none	0=none
1=limited mucosa	1=minimal	1=epithelial desquamation	1=＜50% submucosa thickness	1=mild
2= mucosa and submucosa	2=mild	2=erosion of the epithelial surface	2= （ 50-100% ） submucosa thickness	2=moderate
3=submucosa	3=moderate	3= epithelial surface severe disrupted	3=(100-200%) submucosa thickness	3=marked
4=transmural	4=marked	4=ulceration	4= ＞ 200% submucosa thickness	
