# Supplementary material for: IL-23 Contributes to Campylobacter jejuni-Induced Intestinal Pathology via Promoting IL-17 and IFNγ Responses by Innate Lymphoid Cells
Source: Front Immunol. 2021 Jan 6;11:579615. doi: 10.3389/fimmu.2020.579615 (PMC7815532; doi:10.3389/fimmu.2020.579615)
Supplement: Supplementary Table 2 — Primers for quantitative PCR. [file Table_2.pdf]

Supplementary Table 2. Primers for quantitative PCR

Ifng FW	TCAAGTGGCATAGATGTGGAAGAA
Ifng RV	TGGCTCTGCAGGATTTTCATG
Il17a FW	GGACTCTCCACCGCAATGA
Il17a RV	GGCACTGAGCTTCCCAGATC
Tnf FW	ACGGCATGGATCTCAAAGAC
Tnf RV	AGATAGCAAATCGGCTGACG
Il22 FW	TCCGAGGAGTCAGTGCTAAA
Il22 RV	AGAACGTCTTCCAGGGTGAA
Il12b FW	ACAGCACCAGCTTCTTCATCA
Il12b RV	TCTTCAAAGGCTTCATCTGCAA
Il12a FW	ACAGCACCAGCTTCTTCATCAG
Il12a RV	TCTTCAAAGGCTTCATCTGCAA
Il6 FW	ACAAGTCGGAGGCTTAATTACACAT
Il6 RV	AATCAGAATTGCCATTGCACAA
Il1B FW	AAGCCTCGTGCTGTCGGACC
Il1B RV	TGAGGCCCAAGGCCACAGGT
Il23R FW	GGTCTTCTTGGCCATCATGT
Il23R RV	AGCCACTTTGGGATCATCAG
Hprt FW	GCGTCGTGATTAGTGATGATGAAC
Hprt RV	GAGCAAGTCTTTTCAGTCCTGTCCA
